# Plasticizers and Cardiovascular Health: Role of Adipose Tissue Dysfunction

**DOI:** 10.3389/fphar.2020.626448

**Published:** 2021-02-25

**Authors:** Mikyla A. Callaghan, Samuel Alatorre-Hinojosa, Liam T. Connors, Radha D. Singh, Jennifer A. Thompson

**Affiliations:** ^1^ Department of Physiology and Pharmacology, University of Calgary, Calgary, AB, Canada; ^2^ Libin Cardiovascular Institute, Calgary, AB, Canada; ^3^ Alberta Children’s Health Research Institute, Calgary, AB, Canada

**Keywords:** bisphenols (BPs), phthalates (PAEs), adipogenesis, adipose tissue, cardiovascular disease

## Abstract

Since the 1950s, the production of plastics has increased 200-fold, reaching 360 million tonnes in 2019. Plasticizers, additives that modify the flexibility and rigidity of the product, are ingested as they migrate into food and beverages. Human exposure is continuous and widespread; between 75 and 97% of urine samples contain detectable levels of bisphenols and phthalates, the most common plasticizers. Concern over the toxicity of plasticizers arose in the late 1990s, largely focused around adverse developmental and reproductive effects. More recently, many studies have demonstrated that exposure to plasticizers increases the risk for obesity, type 2 diabetes, and cardiovascular disease (CVD). In the 2000s, many governments including Canada, the United States and European countries restricted the use of certain plasticizers in products targeted towards infants and children. Resultant consumer pressure motivated manufacturers to substitute plasticizers with analogues, which have been marketed as safe. However, data on the effects of these new substitutes are limited and data available to-date suggest that many exhibit similar properties to the chemicals they replaced. The adverse effects of plasticizers have largely been attributed to their endocrine disrupting properties, which modulate hormone signaling. Adipose tissue has been well-documented to be a target of the disrupting effects of both bisphenols and phthalates. Since adipose tissue function is a key determinant of cardiovascular health, adverse effects of plasticizers on adipocyte signaling and function may underlie their link to cardiovascular disease. Herein, we discuss the current evidence linking bisphenols and phthalates to obesity and CVD and consider how documented impacts of these plasticizers on adipocyte function may contribute to the development of CVD.

## Introduction

Cardiovascular diseases (CVD) lead to an estimated 17.9 million deaths annually, making them one of the leading causes of death worldwide.^1^ The substantial global impact of CVD is one of the most critical public health issues of our time. One of the strongest predictors of CVD is obesity. While obesity is considered an independent risk factor for CVD, it frequently occurs in conjunction with other risk factors, including hypertension, insulin resistance and dyslipidemia (Kachur et al., 2017), in what is known as the metabolic syndrome (MetS). Presence of the MetS increases the risk of death from CVD by approximately 2-fold (Ju et al., 2017).

Given that obesity is a major driver of CVD, preventative strategies depend on an understanding of the environmental and socioeconomic factors that underpin worldwide trends in obesity rates. According to the World Health Organization (WHO), over 1.9 billion adults were overweight (BMI > 20) and 650 million adults were obese (BMI > 30) in 2016, representing 39 and 13% of the world population, respectively. If trends continue, approximately half of U.S. adults will be obese by 2030, with one in four experiencing severe conditions (Ward et al., 2019). Over the past decades, the rates of obesity have risen faster in children than in adults (Biro et al., 2016), leading to the present reality that one third of North American children suffer from one or more risk factors for CVD (Tremblay and Willms, 2000; Biro et al., 2016). Approximately three-quarters of overweight or obese children will be obese as adults and at risk for cardiovascular complications (Ward et al., 2019).

The high incidence of obesity is attributable to multiple environmental and lifestyle factors, most notably changes in food production and supply and reductions in physical activity. While consumption of high caloric foods and sedentary behaviour are indeed driving forces in the pathogenesis of cardiometabolic disease, there is some evidence to suggest that they do not fully explain the obesity epidemic (Huo et al., 2016). In the early 2000s, Paula Baillie Hamilton synthesized ecological data to reveal a correlation between increasing rates of obesity in the United States and increasing production of synthetic chemicals (Baillie-Hamilton, 2002). This observation coincided with the emerging theory of endocrine disruption that attributes the homeostasis-disrupting effects of exogenous chemicals to an interference with the synthesis, release, transportation, metabolism, or elimination of endogenous bodily hormones. In 2006, Grun and Blumberg coined the “environmental obesogen hypothesis”, proposing a causal link between environmental toxins and the obesity epidemic (Grün and Blumberg, 2006).

Endocrine disrupting chemicals (EDCs) interfere with hormone signaling by mimicking endogenous ligands to nuclear receptors and acting as agonists or antagonists depending on the dose, species, and cell-type. Plasticizers are among the most pervasive EDCs owing to their high production, slow degradation and leaching into the environment. There are two main groups of plasticizers: 1) bisphenols, which confer rigidity to hard polycarbonate plastics and 2) phthalates, which provide flexibility to soft plastics and polyvinyl chloride (PVC) products. A large body of evidence indicates that these plastics interfere with adipocyte differentiation and adipose tissue function. Since adipose tissue is a critical regulator of cardiovascular health, the effects of plasticizers on adipocyte biology may underlie their association with obesity and CVD. Thus, this review will discuss bisphenols and phthalates, their relationship with MetS, and their impact on adipose tissue development and function.

## Plasticizers

### Bisphenols

Bisphenols are one of the most commonly produced synthetic chemicals worldwide. They are used in the manufacturing of polycarbonate plastics and epoxy resin coatings in food and beverage containers. Additional products containing bisphenols include medical and dental devices, building materials, thermal receipt paper, and children’s toys (Chen et al., 2016). The most common and well-known bisphenol is 2,2-bis(4-hydroxyphenyl) propane or bisphenol A (BPA). Worldwide production of BPA increased by approximately 2.3 million tons between 2003 and 2011 (Flint et al., 2012) and its consumption is expected to increase at a rate of 3.6% per year through 2023^2^. Human exposure primarily occurs through the ingestion of food, beverages and drinking water that have been contaminated through leaching due to incomplete polymerization or polymer degradation. A study conducted on Harvard students found that after a washout period during which BPA exposure was limited, 1 week of drinking from polycarbonate water bottles increased urinary BPA levels by almost 70% (Carwile et al., 2009). In National Health and Nutrition Examination Survey (NHANES) participants that consumed one or more canned foods over a 24 h period, urinary BPA levels were over 50% higher than those who consumed no canned goods (Hartle et al., 2016). Bisphenols can enter the body through routes other than ingestion (Stojanoska et al., 2017) as they are ubiquitous in our environment, detected in surface water, biosolids, soil and air (Corrales et al., 2015).

Ingested BPA is quickly conjugated in the liver and excreted in bile or urine, with an approximate half-life of 6 h (Völkel et al., 2008; Genuis et al., 2012). Despite its rapid metabolism and clearance, BPA is persistent in our environment and detected in over 92% of urine samples (Calafat et al., 2008). Findings of a recent study that employed a new direct method of measuring levels of BPA and its conjugated metabolites suggest that traditional indirect methods used by regulatory bodies to estimate health risk in humans may have underestimated exposure by over 40-fold (Gerona et al., 2020). Studies show that bisphenols cross the placenta and accumulate in fetal tissues at levels higher than maternal serum (Ikezuki et al., 2002; Gerona et al., 2013). This may be due to immature detoxification defences, leading to slower clearance of bisphenols from the fetal compartment, as demonstrated by studies in pregnant sheep (Corbel et al., 2015; Gingrich et al., 2019). The fetus is particularly vulnerable to the endocrine disrupting effects of bisphenols and other xenobiotics as it is undergoing critical developmental stages of organ maturation and setting of endocrine axes.

By 2005, there were over 100 studies showing adverse effects of BPA at or below the safety standard, conducted by dozens of laboratories in the United States, Japan, and Europe. In 2008, the Government of Canada declared BPA a toxic substance and in 2010 banned all import and sales of baby products containing BPA^3^, actions that were followed by the European Union in 2011 and the FDA in 2012. These policies, founded on developments in toxicology and toxicokinetic data, prompted consumer concern that pressured industries to replace BPA with chemical substitutes. BPA analogues share two hydroxyphenol functionalities (Chen et al., 2016). Bisphenol S (BPS), bisphenol F (BPF) and bisphenol AF (BPAF), are the most common analogues and are found in products labeled “BPA-free” (Rochester and Bolden, 2015). Increased production and consumption of BPA analogues have resulted in a rise in environmental and human exposure. Based on data from NHANES, BPA, BPS, and BPF, were detected in 96, 84, and 67% of U.S. adult urine samples, respectively (Lehmler et al., 2018). Another study by Liao et al. reported the presence of BPS in 81% of urine samples collected in the United States (Lehmler et al., 2018). Wang et al. determined that exposure levels of BPA analogues vary across countries, likely a reflection of manufacturing practices or sources of exposure. Human BPS daily intake was highest in Saudi Arabia, France, and Vietnam, whereas human BPF daily intake was highest in Saudi Arabia, the Netherlands and Canada (Wang et al., 2020). Canada, the country that first restricted the use of BPA, had the lowest intake of BPA, but the highest intake of BPF (Wang et al., 2020). While there has been extensive investigation into the health effects of BPA, relatively few studies have explored the analogues that have replaced it. The toxicity of BPA analogues was not investigated sufficiently before introduction to the market, and the data that is available indicate that they exhibit similar endocrine disrupting properties and may lead to the same adverse health effects.

### Phthalates

Phthalates are diesters of 1,2-benzendicarboxylic acid that are used as plasticizers in polymer products, softeners in PVC plastics and fragrance stabilizers in hygiene and cosmetic products (Stojanoska et al., 2017; Wang et al., 2019). Exposure to phthalates is pervasive as they are found in numerous consumer products ranging from adhesives, detergents, automotive plastics, clothing, storage containers, and personal-care products. Human exposure primarily occurs through ingestion, inhalation, or by skin absorption as phthalates can migrate out of products into food, air, dust, and water (Wang et al., 2019). Approximately 60% of ingested phthalates are metabolized within 24 h and excreted in urine; however, metabolites have been detected in blood, saliva, amniotic fluid, and breast milk (Stojanoska et al., 2017).

Di(2-ethylhexyl)phthalate (DEHP) is a high molecular weight phthalate that is most commonly found in plastics and is transformed into several different metabolites after entering the body. Primary monoester metabolites of DEHP include: mono(2-ethylhexyl)phthalate (MEHP), di-n-octyl phthalate (DnOP), di-n-butyl phthalate (DnBP), benzyl butyl phthalate (BBzP), and diethyl phthalate (Lang et al., 2008). Secondary oxidation metabolites include: mono-2-ethyl-5-hydroxyhexyl phthalate (MEHHP), mono-2-ethyl-5-oxohexyl phthalate (MEOHP), and mono-2-ethyl-5-carboxypentyl phthalate (MECPP) amongst many others (Meeker et al., 2012). According to NHANES 1999–2000 data, MEHP was detectable in the urine of >75% participants, and MEP, MBP, and MBzP were detectable in >97% of participants in the USA (Silva et al., 2004). In the Canadian Health Measures Survey 2007–2009, 11 metabolites were monitored, and results indicated that MEP, MnBP, MBzP, MCPP, MEHP, MEOHP, and MEHHP were detected in >90% of Canadians (Saravanabhavan et al., 2013).

In the late 1990s, concerns arose about adverse effects of phthalates in humans, originally focused on DEHP and DINP and their possible reproductive and developmental toxicity. Panels formed by the American Council on Science and Health (ACSH) and the NTP Center for the Evaluation of Risk to Human Reproduction (NTP-CERHR) evaluated the toxicity of a number of phthalates (Kamrin, 2009). In 2008, the US Consumer Product Safety Improvement Act (CPSIA) named limits on the use of six phthalates in children’s products. Under this act DEHP, DBP, and BBP are restricted to an individual concentration limit of 1000 ppm in children’s toys and products for those under the age of 3 (Smith et al., 2020). Whereas, DINP, DIOP, and DnOP are limited to concentrations no greater than 1000 ppm in children’s toys that are small enough to enter a child’s mouth, and in products for those under the age of three (Smith et al., 2020). Canadian and European governments have implemented similar restrictions on these six phthalates. The Chronic Hazard Advisory Panel convened in 2010 recommended further action by US agencies to widen restrictions for DBP, BBP, and DEHP to include additional consumer products. These regulations initiated a push toward safer alternatives, motivating some companies to voluntarily use substitutes with presumed lower toxicity.

## Exposure to Plasticizers and Risk for Metabolic Syndrome: Epidemiological Evidence

### Bisphenols

To-date epidemiological research examining the association between urinary bisphenol concentrations and the development of obesity and other risk factors for CVD have primarily focused on BPA. Many of these studies have been conducted in a cross-sectional design, mainly utilizing data from NHANES (LaKind et al., 2012). Using 2003–2008 data, researchers determined that higher urinary BPA levels were strongly associated with weight circumference (WC) and BMI in males and females over 20 years of age (Shankar et al., 2012). Cai et al. used NHANES 2003–2014 data to determine that higher levels of BPA were associated with increased total CVD burden in males; however, no results were determined in female groups (Cai et al., 2020). Through analysis of 2003–2004 data, Lang et al., similarly demonstrated that CVD was associated with relatively high levels of BPA compared to lower quartiles and that an increase in CVD was associated with a one standard deviation increase of BPA (Lang et al., 2008). While the above findings indicate a relationship between bisphenols and MetS, there has been debate regarding analytical methods and suitability of NHANES data to determine associations. By applying different inclusion criteria, methods and case definitions, Lankind et al. was unable to find associations between BPA concentrations and CVD in multiple NHANES datasets (LaKind et al., 2012).

Data obtained from cohorts other than NHANES provide further evidence to support a relationship between bisphenol exposure and MetS. In a cross-sectional study, Wang et al. analyzed a population of adults over the age of 40 from a community in Shanghai, China (*n* = 3,390). Positive associations between the highest quartiles of BPA exposure and insulin resistance, as well as both general and abdominal obesity were determined (Wang et al., 2012). A case-control study performed by Duan et al. revealed a positive correlation between urinary BPS or BPAF concentrations and type 2 diabetes (T2D) (Duan et al., 2018). Another study aimed to determine the risk of developing T2D over a 9-year period in the French cohort called “Data from an Epidemiological Study on the Insulin Resistance Syndrome” (D.E.S.I.R). Of 755 participants, 201 cases of diabetes were diagnosed, and results suggested that participants in higher quartiles of BPA exposure had nearly double the risk of developing T2D (Rancière et al., 2019). Overall, available data provide strong evidence supporting a link between bisphenol exposure and MetS.

### Phthalates

Several epidemiological studies have explored the relationship between phthalates exposure and the risk for obesity and related metabolic disorders. Using data from NHANES (1992–2002), two studies highlighted a relationship between urinary phthalate metabolites and obesity. Hatch et al. found that BMI and WC were positively associated with exposure to six phthalates in males aged 20–59: the strongest associations occurring with MBzP, MEHHP, and MEOHP (Hatch et al., 2008). However, only MEP significantly predicted BMI and WC in adolescent females, but not in adult females. Stahlhut et al. reported links between MBzP, MEHHP, MEOHP, and MEP and WC (Stahlhut et al., 2007). Both studies determined that MEHP was not significantly correlated with WC, with a possible explanation being its shorter half-life compared to the other studied metabolites.

A cross-sectional study using data from the 2012–2014 Korean National Environmental Health Survey II (*n* = 5,251) reported a significant association between urine MEHHP levels and MetS, defined by NCEP ATP III criteria (Shim et al., 2019). In agreement with these results, James-Todd et al., used NHANES data from 2001 to 2010 (*n* = 2,719) and found that higher concentrations of DEHP metabolites, including MEHP, MEHHP, and MEOHP, increased the odds of developing MetS in males (Shim et al., 2019). Similar to findings revealed by Hatch et al., (2008), no correlations were found in adult females. Gaston and Tulve performed a cross-sectional study with NHANES data from 2003 to 2013 in U.S. adolescents (*n* = 918) and discovered a strong association between MnBP and MetS (Gaston and Tulve, 2019). A smaller study examining MetS patients in a hospital in Prague (*n* = 168) revealed significantly higher urine levels of four phthalate metabolites (MnBP, MEHHP, MEOHP, MECPP) in T2D patients compared to non-diabetic patients, but no relationship with hypertension or dyslipidemia (Piecha et al., 2016). Similarly, another study noted significantly elevated concentrations of DEHP and MECPP in T2D Mexican women; however, correlations between DEHP and IR were only noted for non-diabetic patients (Svensson et al., 2011). Lastly, Huang et al. (2014) determined there was a significant correlation between MnBP, MiBP, MCPP, and DEHP with IR, glycemia, and insulinemia (Huang et al., 2014). In summary, current literature supports a relationship between phthalate exposure and MetS.

## Understanding the Link Between Plasticizers and CVD: Role of Adipose Tissue

Adipose tissue is thought to be a major target for the adverse developmental and functional effects of plasticizers and other EDC as it tends to sequester lipophilic toxins. Numerous investigations have implicated adipose tissue dysfunction as central in the development of obesity-associated CVD. The metabolic consequences of adipose tissue dysfunction, which include insulin resistance, dyslipidemia and increased visceral adiposity among others, are defining features of MetS.

### Adipogenesis

Adipose tissue is the body’s largest endocrine organ and major energy reservoir (Berry et al., 2013). There is growing appreciation for the importance of the “quality” of adipose tissue, over its mass-based quantity, in carrying out its role in regulating systemic metabolic homeostasis (Ikeoka et al., 2010; Akoumianakis et al., 2017). As the body’s main energy reserve, adipose tissue undergoes dynamic remodeling to expand or contract in response to fluctuations in energy balance (Chait and den Hartigh, 2020). In a state of prolonged positive energy balance, subcutaneous fat depots serve as a “metabolic sink” that buffers the excess energy. Healthy expansion of adipose tissue depends on a dynamic balance between hypertrophic growth of existing adipocytes and hyperplastic growth that increases the number of adipocytes through adipogenesis (Chatterjee et al., 2014; Choe et al., 2016; Jeffery et al., 2016). Adipogenesis is the process by which adipocyte stem cells commit and differentiate into mature, lipid-storing adipocytes. When adipogenesis is insufficient, expansion relies on hypertrophy, which beyond a threshold leads to lipid spillover into the circulation and engorged adipocytes that are hypoxic, inflamed and resistant to the anti-lipolytic effects of insulin (Kim et al., 2015; Jang et al., 2016). Thus, failed expansion of adipose tissue underlies the insulin resistance, hyperlipidemia and low-grade inflammation that triggers obesity-induced onset of CVD (Medina-Gomez et al., 2007; Chatterjee et al., 2014).

In adult depots, new adipocytes are recruited from a resident population of progenitors that are committed *in utero*, as revealed by seminal studies by the Gaffe group (Jiang et al., 2014). Therefore, a perturbation in the critical *in utero* window of adipocyte lineage commitment will not only influence postnatal fat mass but may also have later-life consequences for availability of preadipocytes for differentiation and thereby the buffering capacity of adipose tissue. Adipogenesis *in vitro* is increased in response to BPA, as supported by a large body of evidence (Sargis et al., 2010; Boucher et al., 2014; Ohlstein et al., 2014; Ariemma et al., 2016). Much less is known regarding the effect of BPA substitutes on *in vitro* adipogenesis, but the evidence to-date points to similar pro-adipogenic properties. A non-monotonic response to BPS exposure, where increased adipogenesis was observed at lower doses, was reported in stem cells isolated from the subcutaneous depots of female donors (Boucher et al., 2016). In 3T3-L1 murine fibroblasts, pro-adipogenic effects were more pronounced after treatment with BPS compared to BPA (Ahmed and Atlas, 2016). Using the same cell line, a recently published study showed that the adipogenic response of BPS, BPF and BPB occurred at lower doses than that of BPA (Ramskov Tetzlaff et al., 2020). The molecular pathways mediating bisphenol-induced potentiation of adipogenesis are unclear, although a few studies have demonstrated the involvement of estrogen (Boucher et al., 2014) or glucocorticoid (Sargis et al., 2010) signaling.

Phthalates and their metabolites have been studied far less compared to BPA with respect to their effect on preadipocyte differentiation; however, existing evidence indicate similar pro-adipogenic properties. Feige et al. showed increased differentiation via PPARγ activation in 3T3-L1 cells exposed to MEHP, a monoester metabolite of DEHP (Feige et al., 2007). In agreement, a more recent study reported that MEHP promoted differentiation in the same cell line (Qi et al., 2019). Work by Pomatto et al. assessed four plasticizers (DiNP, DiDP, DEGDB, and TMCP) commonly used in the manufacture of food packaging as substitutes for the phthalate DEHP. All DEHP substitutes increased adipogenesis in 3T3-L1 cells, albeit with a maximal response lower than BPA (Pomatto et al., 2018). Another study reported an increase in 3T3-L1 differentiation in response to prolonged exposure to the DEHP substitute, DiNP, an effect that was prevented by PPARγ antagonism (Zhang et al., 2019). Overall, these findings suggest that phthalates and their substitutes augment *in vitro* differentiation of adipocyte progenitors.

While the pro-adipogenic effects of plasticizers in isolated stem cells are well-documented, whether this translates to increased *in vivo* adipogenesis during critical developmental windows of adipose tissue development remains unclear. In offspring born to pregnant rats treated with a low dose of BPA during pregnancy, body weight of both sexes was increased at birth and at weaning total mass and adipocyte size was increased in fat depots of females only (Somm et al., 2009). However, the authors studied only visceral fat, which contributes negligibly to total fat mass in rodents at weaning, as these secondary depots develop primarily after birth (Wang and Scherer, 2014). Later in postnatal life, there were no differences in body weight between offspring born to BPA or vehicle treated dams; however, BPS-exposed offspring were more vulnerable to diet-induced weight gain (Somm et al., 2009). Mice and rats are not ideal species to study the effect of *in utero* exposures on adipogenesis as they are born with very little fat compared to humans, sheep, and guinea pigs. In ovine fetuses of BPA-exposed, but not BPS-exposed dams, there was a sex-dependent increase in differentiation of isolated preadipocytes, without changes in body weight and perirenal adipocyte size (Pu et al., 2017). However, exposure was restricted to mid-gestation (Gd 30–100) despite the accumulation of fat mass occurring predominately in late gestation in sheep and other precocious species. While some studies have examined prenatal bisphenol exposure, fewer have investigated the impact of intrauterine phthalate exposure on early life fat accumulation. One study found higher body weight and visceral adiposity in 8-week old offspring born to pregnant C57BL/6J mice dams exposed to a low dose of the DEHP metabolite, MEHP (Hao et al., 2012).

In humans, studies regarding the relationship between plasticizer exposure and early-life fat mass have yielded inconsistent results. As far as bisphenols, some studies have reported a negative association between maternal exposure and birth weight (Miao et al., 2011; Troisi et al., 2014), while others have found a positive association (Lee et al., 2014). A study by Vafeiadi et al. studied a cohort of 1,363 pregnancies in Greece and showed maternal urinary BPA levels in the first trimester to be negatively associated with BMI in girls between the ages of 1–4, but positively associated with BMI in boys (Vafeiadi et al., 2016). The same study showed that urinary BPA levels were lower in mothers compared to their children and that BPA levels in children at 4 years of age predicted higher BMI and prevalence of obesity. BPA levels in spot urine samples collected from a smaller cohort of pregnant women were negatively associated with BMI in 9 year old girls, with no effect on boys, while BPA levels in children of both sexes were higher in those with greater BMI (Harley et al., 2013). In a Spanish cohort, prenatal BPA levels had no effect on growth in the first 6-months, but was correlated to higher WC and BMI at 4 years of age (Valvi et al., 2013). Overall, these findings suggest that obesity is associated with postnatal rather than prenatal exposure to bisphenols.

Similar to data on bisphenols, current evidence does not support a relationship between prenatal phthalate exposure and birth weight (Shoaff et al., 2016; Chiu et al., 2018). Weight gain in the first 6 months and BMI between ages 1 and 7 were positively associated with maternal DEHP metabolites measured in the first and third trimester, while higher *in utero* exposure decreased early life weight gain in boys (Valvi et al. 2015). No relationship between maternal exposure to DEHP metabolites and fat mass in children aged 4–9 was reported by Buckley et al., (2016b). In a pooled analysis of three cohorts, prenatal exposure to MCPP, a non-specific metabolite of high molecular weight phthalates, was associated with a 2-fold increase in childhood obesity, while exposure to metabolites specific to DEHP was inversely related to childhood obesity (Buckley et al., 2016a). The effect of childhood exposure on adiposity is clearer, with studies showing high levels, particularly in low molecular weight phthalates, to predict childhood obesity (Hatch et al., 2008; Trasande et al., 2013; Deierlein et al., 2016). Together, the above studies underscore the importance of timing of exposure in relation to stages of development. Slower clearance in the fetus due to immature detoxification defences may shift the non-monotonic dose response curve to the right and additionally, toxic effects on the placenta may adversely affect fetal growth. Further, low dose effects are difficult to extract from epidemiological studies due to ubiquitous exposure. As well, studies typically treat EDCs in isolation when human exposure occurs in mixtures. In summary, while *in vitro* studies demonstrate a pro-adipogenic effect of plasticizers, more studies are needed to determine if accelerated fat accumulation due to early life exposure leads to the development of obesity and its cardiometabolic complications.

### Production of Adipokines

Adipose tissue regulates systemic metabolic homeostasis in part through secreting adipokines, a group of adipocyte-derived hormones, proteins, and cytokines with autocrine, paracrine and endocrine effects on energy balance, lipid and glucose metabolism, appetite, insulin sensitivity and inflammation (Ahima and Lazar, 2008). Dysregulated adipokine secretion is a hallmark of hypertrophic adipocyte dysfunction and contributes to the pathogenesis of obesity-associated CVD.

Many plasticizers can alter adipose function by disrupting endocrine signaling in adipose tissue. Obesogenic effects leading to adipocyte hypertrophy and dysfunction may account for dysregulated adipokine release, or EDC may directly influence the endocrine function of adipose tissue. In human adipose tissue explants, treatment with BPA inhibited the release of the hormone adiponectin when present at nanomolar concentrations (Hugo et al., 2008). Adiponectin itself is a 30 kDa protein with the capacity to form several multimers, the synthesis of which is regulated by PPARy receptors (Trujillo and Scherer, 2006). Once released from adipocytes, the physiological effects of adiponectin vary based on the specific adiponectin multimer and tissue-specific receptor to which the protein binds. For example, adiponectin increases fatty acid oxidation and glucose metabolism in muscle when bound to skeletal AdipoR1. When bound to hepatic AdipoR2, however, adiponectin stimulates increased insulin sensitivity. Adiponectin also has the potential to stimulate anti-inflammatory and antiatherogenic effects and is considered to be a key regulator of insulin sensitivity (Trujillo and Scherer, 2006). BADGE, a synthesis product of BPA, has been shown to antagonize PPARy receptors, potentially inhibiting adiponectin expression through this mechanism (Wright et al., 2000). Moreover, BPA may directly inhibit adiponectin synthesis by disrupting the action of protein disulfide isomerase, an enzyme crucial to the assembly and retention of adiponectin (Hiroi et al., 2006). Other bisphenols, including BPF, have also been shown to inhibit adiponectin production (Rochester and Bolden, 2015). Further, the phthalate DEHP has been shown to inhibit the expression of adiponectin in female mice (Schmidt et al., 2012; Klöting et al., 2015). Similarly to bisphenols, this phthalate and its metabolites suppress expression of PPARy receptors (Schmidt et al., 2012).

Plasticizers have also been shown to disrupt the production of the adipokine leptin in adipose tissue, which is a signaling protein involved in regulating feelings of hunger and satiety. BPA exposure was positively associated with serum leptin levels in both humans and rats, although this increase was not correlated with a change in fat mass in human subjects (Wei et al., 2011). Elevation of leptin in these studies was shown to be attributable, in part, to neonatal exposure to BPA (Rönn et al., 2014). The plasticizer, DEHP, a phthalate, has also been shown to increase leptin levels in human pre-adipocytes, although decreased lipid accumulation was observed in this study (Wei et al., 2011; Rönn et al., 2014; Haq et al., 2020). In contrast to the anti-inflammatory properties of adiponectin, leptin stimulates the production of pro-inflammatory cytokines, and an imbalance in the ratio of leptin-to-adiponectin secretion has been associated with obesity and its cardiovascular outcomes (López-Jaramillo et al., 2014).

Adipocytes are responsible for the production of a number of other adipokines, although the effects of plasticizers on these compounds is less studied. Chemerin is a protein produced by adipocytes that is associated with inflammation, metabolic dysfunction and carcinogenesis (Hoffmann et al., 2018). BPA and its halogenated derivatives have been shown to decrease the mRNA expression levels of this peptide in a cancer cell model (Hoffmann et al., 2018). Resistin, an adipokine that interferes with insulin signaling, also shows increased expression *in vitro* in the presence of BPA (Jamaluddin et al., 2012; Menale et al., 2017). The phthalate DEHP did not affect resistin levels in rats, while increases in circulating resistin were observed in female mice after perinatal DEHP exposure (Campioli et al., 2014; Neier et al., 2019). The plasticizer dibutyl phthalate (DBP) has also been negatively correlated with the serum levels of the adipokine omentin (Zhang et al., 2017). The effects of plasticizers on other adipokines such as visfatin and dipeptidyl peptidase 4 have been insufficiently studied. Visfatin, however, is regulated by PPARγ signalling (Choi et al., 2005). Given the previously discussed effects of plasticizers on these receptors, it is possible to speculate that plasticizers may impact visfatin expression.

### Adipose Tissue Inflammation and Oxidative Stress

Inflammation and oxidative stress are core underlying mechanisms in the progression of adipose tissue dysfunction and CVD. BPA has been shown to stimulate the release of inflammatory adipocytokines, including IL-6 and TNF-α from preadipocytes, adipocytes and macrophages within adipose tissue (Heinrich et al., 2003; Ben-Jonathan et al., 2009). Phthalates such as DEHP have also been shown to stimulate TNF-α in adipose tissue (Campioli et al., 2014). While both cytokines exhibit potent inflammatory effects, IL-6 is a pleiotropic cytokine that has been known to stimulate lipolysis, inhibit lipoprotein lipase, and reduce glucose uptake in the adipose tissue. This cytokine furthermore suppresses adiponectin release (Kamimura et al., 2003). TNF-α stimulates lipolysis in the adipose tissue and suppresses insulin sensitivity by downregulating glucose transporter expression, interfering with insulin signaling, and by inhibiting transcription factors involved in insulin sensitivity (Ben-Jonathan et al., 2009).

An increase in oxidative stress in response to BPA has been reported in several cell types (Gassman, 2017). A relationship between the inflammation induced by BPA and oxidative stress has been demonstrated (Ferguson et al., 2016). In an inflammatory state, immune cells such as macrophages are recruited to the adipose tissue; these cells generate reactive oxygen species (ROS) and nitrogen species that both contribute to chronic inflammation and damage cells. Furthermore, oxidative stress induced by BPA was shown to be essential in the activation of the NOD-like receptor protein 3 (NLRP3) inflammasome in adipose cells (Ahmed and Atlas, 2016). The activation of an inflammatory response by BPA-induced oxidative stress causes the recruitment of additional ROS-generating immune cells to the adipose tissue, leading to a sustained cycle of inflammation and oxidative stress (Meli et al., 2020). Alternatively, BPA may induce the production of ROS directly by inhibiting the action of antioxidant enzymes, including superoxide dismutase, catalase, glutathione reductase (GR), and glutathione peroxidase (GSH-Px) (Meli et al., 2020). Further, BPA exposure leads to ATP depletion, cytochrome c release, loss of mitochondrial mass, and loss membrane potential (Lin et al., 2013). Thus, mitochondrial dysfunction may be both a cause and consequence of BPA-induced oxidative stress. Phthalate exposure has also been associated with oxidative stress in adipose tissue (Schaedlich et al., 2018). It has been hypothesized that phthalate-induced oxidative stress is mediated through the activation of PPAR receptors or through changes in mitochondrial function (Trasande and Attina, 2015). The above evidence highlights oxidative stress and inflammation as important pathogenic mechanisms linking plasticizer exposure to adipose tissue dysfunction and CVD.

## Conclusion

**FIGURE 1:**
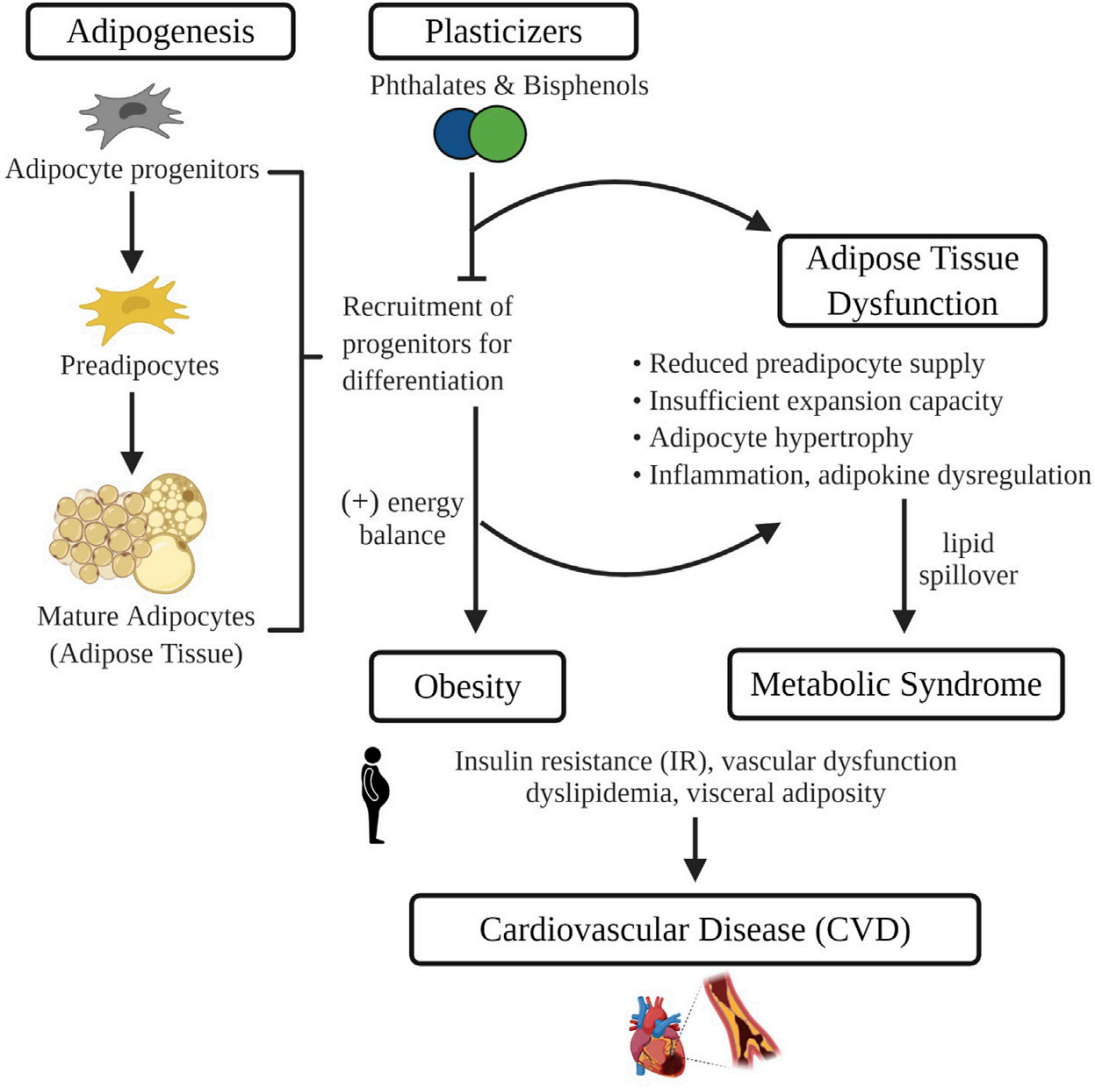
In isolated adipocyte progenitors, differentiation is enhanced with exposure to common plasticizers, phthalates and bisphenols. Therefore, exposure to plasticizers during critical developmental windows of adipogenesis may recruit a greater number of progenitors towards terminal differentiation. Increased adipogenesis during early life leads to the development of obesity and may prematurely deplete the progenitor pool that protects against obesity-associated adipose tissue dysfunction. Adipose tissue dysfunction characterized by an impaired energy buffering capacity, adipocyte hypertrophy and inflammation, is a pivotal pathogenic event in the development of CVD risk factors such as dyslipidemia and insulin resistance. Image made in Biorender.

Commonly used plasticizers, bisphenols and phthalates, are among the most pervasive environmental toxins in our environment. Numerous studies have revealed that exposure to these synthetic chemicals can lead to reproductive and developmental disorders including infertility and early puberty. More recently, exposure has been linked to the pathogenesis of cardiometabolic diseases such as obesity, diabetes and CVD. Given that adipose tissue sequesters environmental toxins and is central to the development of obesity-associated CVD, it may play a critical role in mediating the impact of plasticizers on cardiovascular health (Figure 1). Herein we highlight current evidence surrounding potential mechanisms by which plasticizer exposure modulates adipose tissue development and function. Data described include those from recent studies revealing that synthetic analogues marketed as safer alternatives have similar effects on adipogenesis, oxidative stress and adipose tissue function. These findings emphasize the need for further scientific inquiry into synthetic analogues and their purported safety and continued efforts to limit environmental exposure or develop safer alternatives such as the emerging bio-polymers.

## Footnotes

1
https://www.who.int/health-topics/cardiovascular-diseases#tab=tab_1.

2
https://ihsmarkit.com/products/bisphenol-chemical-economicshandbook.html

3
https://www.canada.ca/en/health-canada/services/chemical-substances/challenge/batch-2/bisphenol-a.html
